# Advertisement to the Faculty, Gentlemen, Farmers, and Graziers, of Pennsylvania, and the United States in General

**Published:** 1818

**Authors:** James Carver


### ADVERTISEMENT

TO THE

### FACULTY,

### GENTLEMEN, FARMERS, AND GRAZIERS,

OF PENNSYLVANIA,

AND THE UNITED STATES IN GENERAL.

---

THE branch of science which I have now the honour to profess in this department of Natural Knowledge, being altogether new in this country, and the *name* by which it is called being but little known, it becomes indispensable, therefore, to communicate, for the better information of the public, whatever may be learned on this head.

*Farriery* is a name which it derived from the occupation of those who practised it, who were in general *Smiths*, or *Workers in Iron* (*Ferracius*). *Veterinary* is a word derived from the Latin, *Veterinarius:* a term appropriated to express either that part of medicine which regards the cure of animals, or the persons who practise that cure. What the true *etymon* of the word may be, is a question of some philological intricacy, though but of little importance. It is sufficient here to say, that the word Veterinarius, as used by Columella and Vegetius, signifies a practitioner in one particular part of medicine; namely, that which respects the cure of diseased cattle; and that art, Veterinaria, signifies the art of healing applied to the healing of cattle.

The word hippiatric, is a compound term, formed of the Greek word *hippos*, a horse, and *cutrace*, medicine, which treats of the cure of diseased horses in particular, and constitutes a principal branch of that division of medicine which treats of the diseases incident to cattle in general and to all other domestic animals.

We have undoubted evidence that the art was cultivated in *very early times.* In the infancy of medicine, when the art of healing was confined to the rude elements of Surgery, it was indiscriminately applied to the relief of all accidental distresses to which the animal frame was liable, whether they occurred in man, or in those animals which constituted his wealth, or were the associates of his labours. In these times, many things occurred to attach the minds of men to the well being of their cattle.

They were almost solely used for tillage, and the dairy; and the life and health of the herds was an especial concern. Cattle was the great medium of exchange, before the invention of coin; and the riches of countries and individuals were estimated by the quantity of cattle and the laws of religion, which religiously forbade the sacrifice of any animal but such as were in the most perfect state of health.

*Chiron* the *Thessalian*, a person whom antiquity held in extreme veneration, and who, from his transcendent skill in horsemanship, and many other useful arts, was called the wise Centaur, lived at the age of the Trojan war. This great man descends to us as the father of medicine, and the instructor of Esculapius in that art. And he was, on the concurrent testimony of antiquity, profoundly skilled therein, as also in the cure and management of cattle.

It would be to no purpose to trace this art minutely through all its vicissitudes; it is sufficient to say that the decline of the Roman empire, and the decay of arts and sciences, occasioned for some time the destruction of *this* as well as every other branch of knowledge. But while Veterinary Medicine was lost in the *West*, and was declining fast in Greece, it found an asylum among the *Arabians;* a nation destined as it should seem by Providence, to receive in trust the knowledge of Europe, until emerged from the abject state into which it was plunged, it was able to reassume its intellectual rank. It is worthy of remark, that the Asiatics appear to have preserved that part of the management of horses, which consists in their treatment when diseased, entirely separate from the business of the farrier; the confusion of which, essentially distinct occupations, has been hitherto the bane of veterinary science among us.—During a residence of fifteen years among the different nations of the East, I have the satisfaction to say I learnt many useful lessons.

The great Lord Bacon, sensible of the services he had rendered to medicine by Zootomy with a view to comparative anatomy, makes the following observation:

" The diligence of Zootomists, says he, may much contribute to illustrate the doctrine of Androtomy, and both inform physicians of the true use of the parts of the human body, and help to decide divers anatomical controversies:—farther, it would be no new thing for naturalists not professedly physicians, to treat of this subject; the naturalist may afford good hints to the practitioners of physic, by trying upon brutes a variety of untried medicaments or remedies, and by suggesting to him both the events of such trials, and also what has been already observed about the cure of diseases incident to beasts.

" The most skilful physicians might also, without disparagement to their profession, do it an useful piece of service, if they would be pleased to collect and digest all the experiments and practices of farriers, graziers, butchers and the like; which the ancients did not despise, but honoured with the title of Hippiatrica and Veterinaria; and among which, if I had leisure, divers things might be taken notice of, which might serve to illustrate this subject."

These are a few of the sentiments of ingenious men, selected of many; but they are sufficient to prove, that from the period at which veterinary medicine first attracted the notice of the learned, it grew more and more an object of their attention.

I shall now follow the progress of this opinion no farther, but observe, that after a course of many years, the government of France undertook to give effectual assistance and protection to this most useful part of Domestic Science, and to provide for it the same advantages by which medicine had formerly advanced.

It will not be out of place to give here some account of the means which the French government employed, in order to bring about the desirable end; and which so justly entitles France to the same honours with respect to the Veterinary Art, which the world must ever concede to the school of Salerno, with respect to medicine.

Sensible of the advantages which must result from such an institution, government granted a sum of 50,000 livres to defray the expenses—providing a laboratory, dispensary, physic, guarding-stables to serve as hospitals, forges, instruments, and utensils; also rooms for study and dissection; in a word, every thing that might render the establishment complete.

The first school was opened in January, 1762. It was very soon filled with native students, and in a short time their numbers were increased by foreigners—supported by the empress queen, the kings of Denmark, Sweden, Poland, Prussia and Sardinia, and the different Swiss Cantons. And within these few years past, Spain, Portugal, Russia,* and all British India, have followed the example.

Dr. Rush, whose heart was ever warm for the introduction of any new branch of science, which might tend to promote the welfare of the animal creation, conversed much with me on veterinary subjects, and laboured hard to prevail on me to establish that pursuit in this city; but not having then obtained it scientifically, I proposed to Dr. Rush and other friends already mentioned, my then intended pursuits at the college—from whence I am now returned and commenced practice.

* The emperor of Russia has lately sent over twelve veterinary surgeons, and twenty-four shoeing-smiths.

